# Efficacy and safety of therapies for EGFR-mutant non-small cell lung cancer with brain metastasis: an evidence-based Bayesian network pooled study of multivariable survival analyses

**DOI:** 10.18632/aging.103455

**Published:** 2020-07-15

**Authors:** Binghao Zhao, Yuekun Wang, Yaning Wang, Wenlin Chen, Lizhou Zhou, Peng Hao Liu, Ziren Kong, Congxin Dai, Yu Wang, Wenbin Ma

**Affiliations:** 1 Departments of Neurosurgery, Peking Union Medical College Hospital, Chinese Academy of Medical Sciences and Peking Union Medical College, Beijing 100730, China

**Keywords:** EGFR-mutant, NSCLC, brain metastasis, Bayesian network pooled study

## Abstract

Preferable treatments for epidermal growth factor receptor (EGFR)-mutant non-small cell lung cancer (NSCLC) with brain metastasis are elusive. The study intended to estimate the relative efficacy and safety of systemic therapies. Clinical trials about therapies for EGFR-mutant, brain-metastatic NSCLC were identified. Progression-free survival (PFS) and overall survival (OS) were analysed using random effects Bayesian network meta-analyses (NMAs) on the hazard ratio (HR)-scale. Nomogram and Kaplan-Meier plots based on clinical or individual factors are displayed using data obtained from the Surveillance Epidemiology and End Results (SEER) database. Third-generation EGFR- tyrosine kinase inhibitors (EGFR-TKI) (osimertinib), EGFR-TKIs + stereotactic radiosurgery (SRS)/whole brain radiotherapy (WBRT) (gefitinib/erlotinib + SRS/WBRT), and EGFR-TKIs (erlotinib) + anti-vascular endothelial growth factor receptor (anti-VEGFR) (bevacizumab) achieved superior PFS (HR: 0.30 (0.15-0.59); HR: 0.47 (0.31-0.72); HR: 0.50 (0.21-1.21) vs. deferring SRS/WBRT) and acceptability; EGFR-TKIs + SRS/WBRT was top ranking (vs. others) for OS followed by third-generation EGFR-TKI. In the dataset cohort of 1173 brain-metastatic NSCLC patients, the 6-month, 1-year, and 3-year survival rates were 59.8%, 41.3%, and 5.6%, respectively. Race and origin, and year of diagnosis were independent predictors of OS. Survival curves showed that the OS of patients varied significantly by histology and race. Third-generation EGFR-TKI and EGFR-TKIs + SRS/WBRT are more effective and potentially acceptable for EGFR-mutant NSCLC with brain metastases balancing OS and PFS. Surgeries without adjuvant therapies cannot significantly improve the OS of brain-metastatic NSCLC patients. The study highlights importance of osimertinib in these patients and provide a reference for clinical treatments.

## INTRODUCTION

Treatment for non-small cell lung cancer (NSCLC) with central nervous system (CNS) metastases includes surgery, stereotactic radiosurgery (SRS), and whole brain radiotherapy (WBRT) [1]. Growing evidence suggests that patients with epidermal growth factor receptor (EGFR) gene mutation-positive NSCLC are quite prone to the development of brain metastases, with the frequency ranging from 44% to 63% [2] (Supplementary Figure 1). For NSCLC patients with EGFR mutations and brain metastases, the standard care includes first- and second-generation EGFR tyrosine kinase inhibitors (EGFR-TKIs), such as gefitinib, erlotinib or afatinib. In fact, using EGFR-TKI can reduce the risk of CNS metastasis compared with chemotherapy or WBRT over the course of disease [3]. In reviewing clinical trials [4–21] on EGFR-mutant, brain-metastatic NSCLC and other relevant-topic studies (on advanced patients), multiple EGFR-TKIs have exhibited clinical benefits of enhanced progression-free survival (PFS) and the objective response rate (ORR) as well as a safer profile on these patients over platinum-based chemotherapy [22–26] and radiotherapy (RT) [27]. Clinical benefits of icotinib were also exhibited [28]. Notably, osimertinib (third generation TKI) is specifically selected for EGFR-TKI sensitive and T790M resistant mutations with better CNS penetration than previous agents, and it has displayed greater efficacy than chemotherapy for T790M-positive NSCLC in phase III AURA3 trial [12]. With these developments, questions regarding the relative efficacy and safety between any two of these multiple treatments have emerged.

The spectrum of therapies for brain-metastatic EGFR-mutant NSCLC patients is wide, spanning multiple treatment classes; however, there is a lack of head-to-head evidence comparing EGFR-TKIs plus RT to newly emerged third-generation TKIs. Moreover, the selection priority is still unclear owing to the lack of standard guidelines, it appears difficult to draw conclusions regarding the effects of individual treatments. This study aimed to (1) provide an up-to-date literature analysis evaluating the efficacy and safety of individual regimens for brain-metastatic EGFR-mutant NSCLC patients and (2) provide more prognostic information about brain-metastatic NSCLC patients based on data from large-population registries.

## RESULTS

### Study search and characteristics of included studies

We identified 521 relevant records from the searched databases (497 from the mentioned databases and 24 from the reference lists of retrieved studies). Thirty-four studies were subjected to full-text review, and 18 studies were included in the Bayesian study (Supplementary Figure 2).

A total of 1710 EGFR-mutant NSCLC patients with brain metastasis in 18 studies [4–21] were randomly assigned to receive one of the following 10 medications classes: platinum-based chemotherapy, first-generation EGFR-TKI, second-generation EGFR-TKI, third-generation EGFR-TKI, EGFR-TKIs + platinum-based chemotherapy, EGFR-TKIs + SRS/WBRT, deferring SRS/WBRT, WBRT, EGFR-TKIs + anti-vascular endothelial growth factor receptor (anti-VEGFR), and EGFR-TKIs + MET-TKIs.

The mean age of the participants was 60.3 y, and the mean trial duration was 3.2 y. No eligible trials had assigned special prior treatment relevant to their investigated interventions in protocol, however, not close-related treating history (chemotherapy-naive, targeted therapy-naive, precautionary regimens etc.) of these patients was reported in several studies [6, 7, 10, 11, 13, 17, 19]. With regard to the outcomes, 16 studies [4, 5, 7–20] (9 treatment classes) reported PFS (Wu et al [18] mainly reported CNS PFS), and 12 studies [4–8, 10, 11, 13–15, 19, 21] (9 classes) reported OS. There were 4 [4, 8, 9, 11] phase II (IIa plus IIb) studies and 14 [5–7, 10, 12–21] phase III studies. Published time of eligible studies was from 2013 [4] to 2019 [20, 21]. Prognostic data were retrieved from survival curves in 2 studies [8, 11]. All these studies were published in English (Table 1).

**Table 1:** Characteristics of included studies.

**Source**	**Period**	**Sex and mean age**	Prior-treatment*	**Arm 1 (number)**	**Arm 2 (number)**	**Outcome†**	**Study design (registration information)**
Sequist et al, 2013 (America) ^[4]^	2009-2011	M/F, 61.6	not assigned	40 mg/d afatinib (20)	75 mg/m^2^ cisplatin + 500 mg/m^2^ pemetrexed every 3 weeks (15)	OS: HR, 1.14 (95% CI, 0.55-2.30); PFS: HR 0.54 (95% CI, 0.23-1.25)	phase II RCT (NCT01121393)
Wu et al, 2014 (multi nations) ^[5]^	2010-2011	M/F, 54.1	not assigned	40 mg/d afatinib (28)	75 mg/m^2^ cisplatin + 1000 mg/m^2^ gemcitabine every 3 weeks (18)	OS: HR, 1.13 (95% CI, 0.56-2.26); PFS: HR 0.47 (95% CI, 0.18-1.21)	phase III RCT (NCT00949650)
Scagliotti et al, 2015 (multi nations) ^[6]^	2011-2012	M/F, 61.2	not assigned, part with EGFR-TKI or MET-TKI-naive regimes history	150 mg/d erlotinib + 720 mg/d tivantinib (56)	150 mg/d erlotinib (53)	OS: HR, 0.72 (95% CI, 0.35-1.48)	phase III RCT (NCT01244191)
Soria et al, 2015 (multinations) ^[7]^	2012-2013	M/F, 59.0	not assigned, part with chemotherapy-naive regimen history, CR or PR after at least 6 months first-line gefitinib	250 mg/d gefitinib + 75 mg/m^2^ cisplatin and 500 mg/m^2^ pemetrexed (44)	75 mg/m^2^ cisplatin and 500 mg/m^2^ pemetrexed every 3 weeks (31)	OS: HR, 1.55 (95% CI, 1.00-2.41); PFS: HR 0.80 (95% CI, 0.61-1.06)	phase III RCT (NCT01544179)
Magnuson et al, 2016 (America) ^[8]^	2008-2014	M/F,59.3	not assigned	upfront 150 mg/d erlotinib followed by SRS/WBRT (17)	upfront SRS/WBRT followed by 150 mg/d erlotinib (33)	OS: HR, 2.48 (95% CI, 1.34-4.60); PFS: HR, 2.13 (95% CI, 1.57-2.69)	phase II RCT (NCT01763385)
Park et al, 2016 (multinations) ^[9]^	2011-2013	M/F, NA	not assigned	40 mg/d afatinid (26)	250 mg/d gefitinib (24)	PFS: HR, 0.73 (95% CI, 0.58-0.92)	phase II b RCT (NCT01466660)
Schuler et al, 2016 (Germany) ^[10]^	2009-2012	M/F, 57.3	not assigned; part with WBRT history	40 mg/d afatinid (48)	75 mg/m^2^ cisplatin + 500 mg/m^2^ pemetrexed or 1000 mg/m^2^ gemcitabine every 3 weeks (33)	OS: HR, 1.14 (95% CI, 0.66-1.94); PFS: HR, 0.50 (95% CI, 0.27-0.95)	phase III RCT (NA)
Fan et al, 2017 (China) ^[11]^	2011-2014	M/F,58.0	not assigned, part with platinum-based chemotherapy history	375 mg/d icotinib + SRS/WBRT (WBRT (46) and SRS (10)) (56)	375 mg/d icotinib alone (41)	OS: HR, 0.47 (95% CI, 0.24-0.95); PFS: HR, 0.63 (95% CI, 0.35-1.14)	phase II RCT (NCT01516983)
Mok et al, 2017 (Multinations) ^[12]^	2014-2016	M/F, 62.3	not assigned	80 mg/d osimertinib (93)	500 mg/m^2^ pemetrexed + carboplatin or 75 mg/m^2^ cisplatin every 3 weeks (51)	PFS: HR, 0.32 (95% CI, 0.21-0.49)	phase III RCT (NCT02151981)
Mok et al (2), 2017 (Multinations) ^[13]^	2012-2015	M/F, NA	not assigned, precautionary premedication regimens (antiemetic, hydration, corticosteroid treatment) were taken to reduce toxicity in platinum group	250 mg/d gefitinib + 75 mg/m^2^ cisplatin and 500 mg/m^2^ pemetrexed (44)	75 mg/m^2^ cisplatin and 500 mg/m^2^ pemetrexed every 3 weeks (31)	OS: HR, 1.31 (95% CI, 0.97-1.77), PFS: HR, 0.79 (95% CI, 0.60-1.05)	phase III RCT (NCT01544179)
Yang et al, 2017 (China) ^[14]^	2012-2016	M/F, 57.5	not assigned	375 mg/d icotinib (85)	WBRT alone 10 fractions (73)	OS: HR, 0.93 (95% CI, 0.60-1.44), PFS: HR, 0.56 (95% CI, 0.36-0.90)	phase III RCT (NCT01724801)
Zhu et al, 2017 (China) ^[15]^	2011-2015	M/F, 56.0	not assigned	250 mg/d gefitinib or 150 mg/d erlotinib (66)	EGFR-TKI + SRS/WBRT (67)‡	OS: HR, 1.82 (95% CI, 1.11-2.98), PFS: HR, 1.62 (95% CI, 1.07-2.45)	phase III RCT (approved by the institutional review board)
Reungwetwattana et al, 2018 (multinations) ^[16]^	2015-2017	M/F, 63.0	not assigned	80 mg/d osimertinib (61)	250 mg/d gefitinib or 150 mg/d erlotinib (67)	PFS: HR, 0.48 (95% CI, 0.26-0.86)	phase III RCT (NCT02296125)
Soria et al, 2018 (multinations) ^[17]^	2015-2017	M/F, 64.0	not assigned, part with definitive treatment or glucocorticoid therapy history§	80 mg/d osimertinib (53)	250 mg/d gefitinib or 150 mg/d erlotinib (63)	PFS: HR, 0.47 (95% CI, 0.30-0.74)	phase III RCT (NCT02296125)
Wu et al, 2018 (multinations) ^[18]^	2014-2016	M/F, 58.0	not assigned	80 mg/d osimertinib (75)	cisplatin or 75 mg/m^2^ carboplatin + 500 mg/m^2^ Pemetrexed every 3 weeks (41)	PFS: HR, 0.32 (95% CI, 0.15-0.69)	phase III RCT (NCT02151981)
Yang et al, 2018 (China) ^[19]^	2013-2016	M/F, NA	not assigned, part with EGFR-TKI and brain RT-naive regimen history	150 mg/d erlotinib + WBRT (55)	WBRT alone (54)	OS: HR, 0.91 (95% CI, 0.68-1.23); PFS: HR, 0.97 (95% CI, 0.74-1.28)	phase III RCT (NCT01887795)
Saito et al, 2019 (Japan) ^[20]^	2015-2017	M/F, 67.5	not assigned	150 mg/d erlotinib + bevacizumab 15mg/kg every 3 weeks (36)	150 mg/d erlotinib (36)	PFS: HR, 0.78 (95% CI, 0.42-1.43)	phase III RCT (UMIN000017069)
Ramalingam et al, 2019 (multinations) ^[21]^	2015-2019	M/F, 64.0	not assigned	80 mg/d osimertinib (53)	250 mg/d gefitinib or 150 mg/d erlotinib (63)	OS: HR, 0.83 (95% CI, 0.53-1.30)	phase III RCT (NCT02296125)

*Essential or preset treatments for participants considering their diseases or conditions. † PFS was the primary endpoint of current study. PFS here actually represented CNS PFS. ‡ EGFI-TKI here included 250 mg/d gefitinib or 150 mg/d erlotinib. § These previous definitive treatment or glucocorticoid therapy had to be completed at least two weeks before launching the trial treatment.

Related EGFR-TKIs (gefitinib, erlotinib, icotinib, afatinib, osimertinib) and MET-TKI (tivantinib) were oral used, platinum-based therapies were prescribed intravenously.

Abbreviations: OS, overall survival; PFS, progression-free survival; iPFS, intracranial progression-free survival; HR, hazard ration; RCT, randomized controlled trial; EGFR, epidermal growth factor receptor; WBRT, whole brain radiotherapy; SRS, stereotactic radiotherapy; EGFR-TKI, epidermal growth factor receptor-tyrosine kinase inhibitor; CR, complete response; PR, partial response; NA, not available.

Detailed methodological quality of the included trials was presented in Supplementary Table 2; one study [11] had unclear random sequence generation; 8 studies had several selection biases; 12 studies were at high risk of bias based on the blinding of participants; and 6 studies [4, 5, 13, 14, 18, 20] were open-label studies. The study by Yang et al [19] was a conference abstract from the IASLC but with useful data.

### Effect on progression-free survival

**Figure 1:**
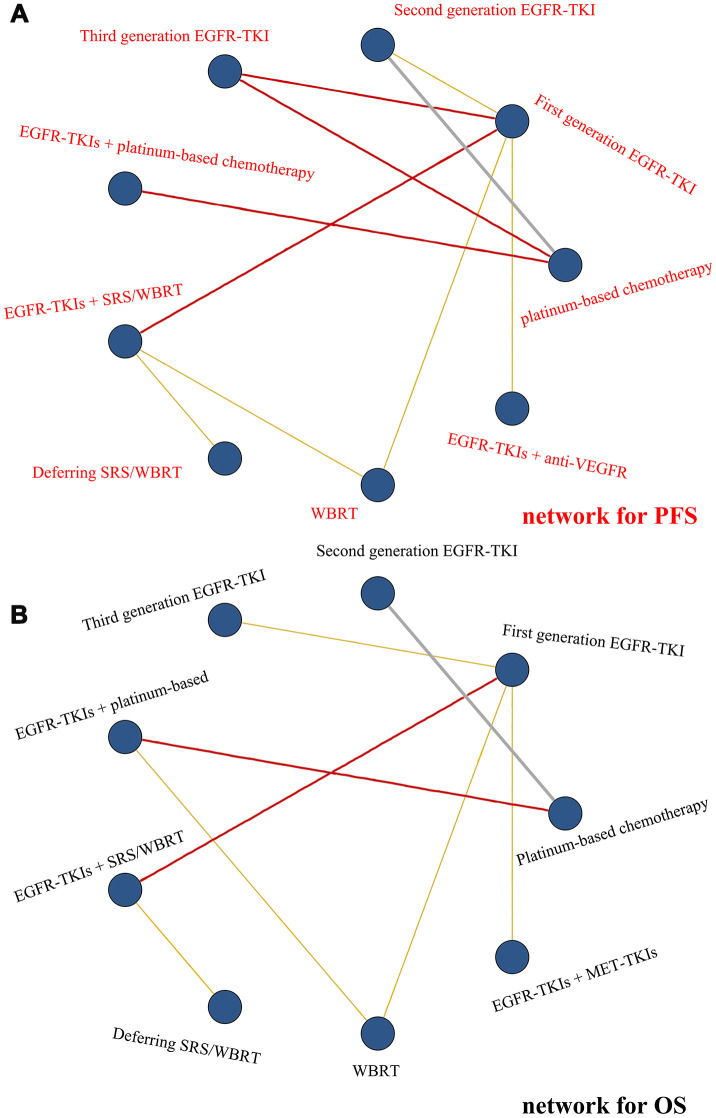
**Networks of comparisons of the multiple treatments with regard to efficacy.** (**A**) Network for PFS and (**B**) network for OS. Each node corresponds to a treatment included in the analysis. Each line corresponds to direct comparisons between treatments with the width corresponding to the number of direct within-trial comparisons. Treatments are listed around each node.

Nine treatment classes for PFS were presented in Figure 1A. The deviance information criteria (DIC) for the random model fit was 21.978. Third-generation EGFR-TKI seemed to be more efficacious than most treatment classes in the network, although compared to EGFR-TKIs + anti-VEGFR (HR, 0.61; 95% Crl, 0.28 to 1.31) as well as EGFR-TKIs + SRS/WBRT (HR, 0.64; 95% Crl, 0.38 to 1.07), it was not statistically significant. However, the levels of efficacy of EGFR-TKIs + SRS/WBRT and second-generation EGFR-TKI were comparable (HR, 1.00; 95% Crl, 0.60 to 1.66) and those of deferring SRS/WBRT and platinum-based chemotherapy were comparable (HR, 1.08; 95% Crl, 0.53 to 2.19). The efficacy of third-generation EGFR-TKI for PFS was significantly the best, followed by EGFR-TKIs + anti-VEGFR, then EGFR-TKIs + SRS/WBRT and second-generation EGFR-TKI, then first-generation EGFR-TKI ranked fourth, EGFR-TKIs + platinum-based chemotherapy ranked fifth, WBRT ranked sixth, and deferring SRS/WBRT and platinum-based chemotherapy tied for seventh. Differences in PFS between therapies within the same treatment class were not statistically significant. The relative treatment effects are shown in Table 2 and Supplementary Figure 3.

**Table 2:** Efficacy of summarized therapies according to multi-treatments Bayesian study (OS+ PFS) (HR with 95% Crl).

Platinum-based chemotherapy	1.41 (0.55-3.72)	1.14 (0.71-1.78)	1.18 (0.38-3.87)	1.39 (0.91-2.16)	0.73 (0.25-2.20)	1.81 (0.49-6.79)	1.52 (0.75-3.11)	NA	1.03 (0.29-3.60)
1.46 (0.94-2.27)	First generation EGFR-TKI	0.80 (0.27-2.27)	0.83 (0.43-1.59)	0.98 (0.42-2.28)	**0.52 (0.31-0.88)**	1.28 (0.49-3.23)	1.07 (0.56-2.01)	NA	0.72 (0.31-1.68)
**1.99 (1.34-2.97)**	1.36 (0.96-1.95)	Second generation EGFR-TKI	1.04 (0.31-3.68)	1.23 (0.67-2.31)	0.64 (0.20-2.16)	1.59 (0.40-6.57)	1.34 (0.59-3.13)	NA	0.90 (0.23-3.41)
**3.10 (2.12-4.55**)	**2.13 (1.47-3.08)**	**1.55 (1.01-2.40)**	Third generation EGFR-TKI	1.18 (0.41-3.42)	0.62 (0.26-1.42)	1.54 (0.50-4.78)	1.29 (0.52-3.20)	NA	0.86 (0.29-2.56)
1.26 (0.93-1.70)	0.86 (0.51-1.47)	0.63 (0.38-1.04)	**0.41 (0.25-0.66)**	EGFR-TKIs + platinum-based chemotherapy	0.53 (0.20-1.40)	1.31 (0.37-4.52)	1.10 (0.62-1.93)	NA	0.74 (0.22-2.37)
**1.98 (1.12-3.47**)	1.36 (0.95-1.95)	1.00 (0.63-1.66)	0.64 (0.38-1.07)	1.58 (0.83-2.96)	EGFR-TKIs + SRS/WBRT	**2.47 (1.14-5.34)**	2.08 (0.91-4.73)	NA	1.40 (0.51-3.78)
0.93 (0.46-1.89)	0.64 (0.37-1.11)	**0.47 (0.24-0.91)**	**0.30 (0.15-0.59)**	0.74 (0.34-1.58)	**0.47 (0.31-0.72)**	Deferring SRS/WBRT	0.84 (0.27-2.62)	NA	0.56 (0.16-2.00)
1.12 (0.60-2.05)	0.77 (0.50-1.17)	**0.56 (0.32-0.97)**	**0.36 (0.21-0.64)**	0.89 (0.44-1.75)	**0.56 (0.37-0.85)**	1.20 (0.67-2.15)	WBRT	NA	0.67 (0.23-1.92)
1.89 (0.82-4.26)	1.28 (0.65-2.54)	0.94 (0.43-2.03)	0.61 (0.28-1.31)	1.49 (0.62-3.53)	0.94 (0.44-2.03)	2.01 (0.83-4.81)	1.68 (0.74-3.72)	EGFR-TKIs + anti-VEGFR	NA
NA	NA	NA	NA	NA	NA	NA	NA	NA	EGFR-TKIs + MET-TKIs

Comparisons among treatments should be read from left to right, the summarized estimate is in each blank in common between the column-indicated treatment and the row-indicated treatment. For these results, HR and 95% Crl less than 1 favors the column-indicated treatment, HR and 95% Crl higher than 1 favors the row-indicated treatment. Red represents treatments; light blue represents outcomes of PFS; light grey represents outcomes of OS. To obtain HRs for comparisons in the opposite direction, reciprocals should be taken. Significant results are in bold and underscored forms.

Abbreviations: OS, overall survival; PFS, progression-free survival; Crl, credible interval; EGFR-TKI, epidermal growth factor receptor-tyrosine kinase inhibitor; RT, radiotherapy; WBRT, whole brain radiotherapy; VEGFR, vascular endothelial growth factor receptor; NA, not available.

Edge-splitting exercise revealed no significant evidence of inconsistency in the network. Comparisons between third-generation EGFR-TKI and first-generation EGFR-TKI (HR, 0.47; 95% Crl, 0.30 to 0.74; *P*_internation for inconsistency_ = 0.066), EGFR-TKIs + SRS/WBRT and first-generation EGFR-TKI (HR, 0.62; 95% Crl, 0.42 to 0.91; *P*_internation_ = 0.066), WBRT and first-generation EGFR-TKI (HR, 1.80; 95% Crl, 1.06 to 3.04; *P*_internation_ = 0.064) were significantly different based on direct evidence rather than indirect evidence; comparison between WBRT and EGFR-TKIs + SRS/WBRT was significantly different on indirect evidence (HR, 2.87; 95% Crl, 1.50 to 5.56; *P*_internation_ = 0.074) rather than direct evidence (Table 3).

**Table 3:** Edge-splitting method for direct and indirect evidence relating to progression-free survival and overall survival

**Multiple-treatment**	Direct comparison outcome*	Indirect comparison outcome*	Combined outcome*†	**P-value‡**
**PFS**				
Second generation EGFR-TKI	0.50 (0.31-0.84)	0.50 (0.22-1.10)	**0.50 (0.34-0.75)**	0.975
Platinum-based chemotherapy				
Third generation EGFR-TKI	0.32 (0.20-0.51)	0.33 (0.14-0.74)	**0.32 (0.22-0.48)**	0.95
Platinum-based chemotherapy				
Second generation EGFR-TKI,	0.73 (0.46-1.15)	0.75 (0.33-1.67)	0.74 (0.51-1.06)	0.959
First generation EGFR-TKI				
Third generation EGFR-TKI	**0.47 (0.30-0.74)**	0.46 (0.21-1.06)	**0.47 (0.33-0.69)**	0.964
First generation EGFR-TKI				
EGFR-TKIs + SRS/WBRT	**0.62 (0.42-0.91)**	1.33 (0.64-2.76)	0.74 (0.51-1.06)	0.066
First generation EGFR-TKI				
WBRT	**1.80 (1.06-3.04)**	0.84 (0.45-1.58)	1.31 (0.85-2.01)	0.064
First generation EGFR-TKI				
WBRT	1.35 (0.83-2.23)	**2.87 (1.50-5.56)**	**1.77 (1.16-2.69)**	0.074
EGFR-TKIs + SRS/WBRT				
**OS**				
Second generation EGFR-TKI	**0.50 (0.31-0.84)**	0.50 (0.22-1.10)	**0.50 (0.34-0.75)**	0.975
Platinum-based chemotherapy				
Third generation EGFR-TKI	**0.32 (0.20-0.51)**	**0.33 (0.14-0.74)**	**0.32 (0.22-0.48)**	0.95
Platinum-based chemotherapy				
Second generation EGFR-TKI	0.73 (0.46-1.15)	0.75 (0.33-1.67)	0.74 (0.51-1.06)	0.959
First generation EGFR-TKI				
Third generation EGFR-TKI	**0.47 (0.30-0.74)**	0.46 (0.21-1.06)	**0.47 (0.33-0.69)**	0.964
First generation EGFR-TKI				
EGFR-TKIs + SRS/WBRT	**0.62 (0.42-0.91)**	1.33 (0.64-2.76)	0.74 (0.51-1.06)	0.068
First generation EGFR-TKI				
WBRT	**1.80 (1.06-3.04)**	0.84 (0.45-1.58)	1.31 (0.85-2.01)	0.064
First generation EGFR-TKI				
WBRT	1.35 (0.83-2.23)	**2.87 (1.50-5.56)**	**1.77 (1.16-2.69)**	0.074
EGFR-TKIs + SRS/WBRT				

* Outcomes are in HR (95% Crl) form. † Combined outcome includes direct comparison plus indirect comparison outcomes. ‡ P-value < 0.05 means there is significant difference between direct comparison and indirect comparison outcomes. Abbreviations: EGFR-TKI, epidermal growth factor receptor-tyrosine kinase inhibitor; SRS, stereotactic radiosurgery; WBRT, whole brain radiotherapy; HR, hazard ration; 95% Crl, 95% credible interval; PFS, progression-free survival; OS, overall survival

### Effect on overall survival

Nine treatment classes for OS were identified in Figure 1B, the DIC for model fitness was 18.370. Treatment with EGFR-TKIs + SRS/WBRT seemed to be superior vs. most of the treatments, although there was only statistical significance compared to first-generation EGFR-TKI (HR, 0.52; 95% Crl, 0.31 to 0.88) and deferring SRS/WBRT (HR, 0.40; 95% Crl, 0.19 to 0.87). The efficacy of third-generation EGFR-TKI and EGFR-TKIs + MET-TKIs (HR, 1.16; 95% Crl, 0.39 to 3.40), platinum-based chemotherapy and second-generation EGFR-TKI (HR, 0.88; 95% Crl, 0.56 to 1.40), first-generation EGFR-TKI and EGFR-TKIs + platinum-based chemotherapy (HR, 1.02; 95% Crl, 0.44 to 2.37) was statistically comparable. EGFR-TKI + SRS/WBRT ranked first in terms of OS, third-generation EGFR-TKI and EGFR-TKIs + MET-TKIs ranked second equally, platinum-based chemotherapy and second-generation EGFR-TKI ranked third equally, EGFR-TKIs + platinum-based chemotherapy and first-generation EGFR-TKI ranked fourth equally, WBRT ranked fifth, deferring SRS/WBRT ranked last. Relative treatment effects are reflected in Table 2 and Supplementary Figure 4.

The edge-splitting method revealed no significant evidence of inconsistency. Second-generation EGFR-TKI vs. platinum-based chemotherapy, third-generation EGFR-TKI vs. platinum-based chemotherapy and first-generation EGFR-TKI were statistically significant based on both direct and overall effect estimates; EGFR-TKIs + SRS/WBRT as well as WBRT vs. first-generation EGFR-TKI were statistically significant only based on direct evidence (Table 3).

### Safety

There was one study [14] that precisely summarized the adverse events of treatments on identified brain-metastatic EGFR-mutant NSCLC patients. Severe adverse events (toxicity grade≥ 3) were found in 7 (8%) and 28 (38%) patients in the icotinib and WBRT groups, respectively. The most common adverse events were gastrointestinal symptoms and CNS disorders. Icotinib (first-generation EGFR-TKI) was likely to have a better safety profile than WBRT. It was implied that platinum-based chemotherapy is more intolerant than targeted therapies.

### Prognosis by gene symbol

In multivariate K-M plots of EGFR-mutant NSCLC patients, we found a significant difference in OS between EGFR-mutant and wild-type individuals in the total population (HR, 0.82; 95% confidence interval (CI), 0.70 to 0.97), restricted to males (HR, 0.74; 95% CI, 0.60 to 0.91), stage I NSCLC (HR, 0.43; 95% CI, 0.30 to 0.60) and adenocarcinoma (HR, 0.78; 95% CI, 0.61 to 1.00) (Supplementary Figure 5). More PFS benefits were found in the stage I population (HR, 0.53; 95% CI, 0.28 to 0.98) (Supplementary Figure 6).

### Individual patient-level analyses

**Figure 2:**
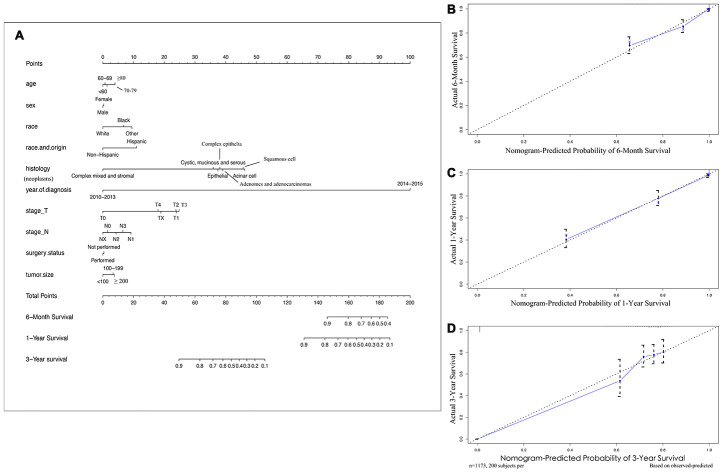
**Nomograms and calibration curves for 6-month, 1-year, 3-year survival rates of NSCLC patients with brain metastases.** (**A**) Survival nomogram (**B**) calibration curve for 6-month survival rate, (**C**) 1-year, (**D**) and 3-year survival rates. To use the nomogram, individual patient data are located on each variable axis, and a line is drawn upward to determine the score received for each potential variable value. The sum of these scores is located on the Total Points axis, and a line is drawn downward to the survival rate axes to discern the likelihood of 6-month, 1- or 3-year survival. The calibration curves were plotted for the primary cohort, in which the nomogram-predicted probability of overall survival is plotted on the X-axis, and real overall survival is plotted on the y-axis. More overlap between the blue lines and dotted lines show the good predictive ability of the nomogram.

**Figure 3:**
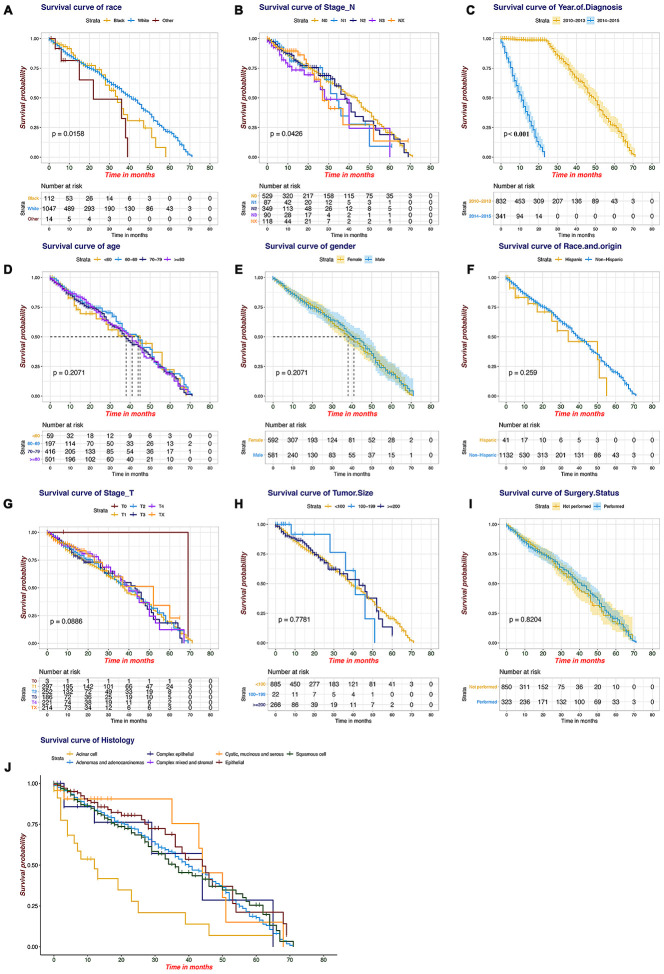
**Kaplan-Meier plots for the survival of the SEER patients.** (**A**) Race, (**B**) stage_N, (**C**) year of diagnosis, (**D**) age, (**E**) gender, (**F**) race and origin, (**G**) stage_T, (**H**) tumour size, (**I**) surgery status, (**J**) histology.

SEER primary cohort incorporated 1173 brain metastatic NSCLC patients with complete individual data were mainly analysed. The median OS was 10 months (range, 1 to 71 months), and the estimated 6-month, 1-year, and 3-year OS rates were 59.8%, 41.3%, and 5.7%, respectively. The multivariable analyses demonstrated that race and origin (P_min_ = 0.037) and year of diagnosis (P_min_ < 0.001) were independent predictors of the OS of metastatic NSCLC patients (Supplementary Table 3). A prognostic nomogram with all investigated predictive factors for OS is shown in Figure 2A. The C-index for prediction (0.847, 95% CI: 0.829 to 0.864), and the calibration plots for the probability of survival at 6 months, 1 year, and 3 years displayed an optimal agreement between the nomogram prediction and actual results (Figure 2B–2D). K-M plots for individuals were shown in Figure 3, which illustrated that the log-rank P value appeared to vary over categories of each factor. There were significant survival differences based on histology (P < 0.001) and race (P = 0.016) (P value of stage_N was so close to 0.05); cystic, mucinous and serous neoplasms had more significant OS benefits (median OS 9 months) based on histology, and white patients appeared to have longer OS (median OS 10 months) than those of other races. It is recommended to record data pertaining to targeted biomarkers in future datasets.

## DISCUSSION

The recent evolution of targeted therapies, RT, and immunotherapy has led to a wide range of treatment alternatives for metastatic NSCLC with EGFR mutations. We derived several principal findings: third-generation EGFR-TKI (osimertinib) and EGFR-TKIs + SRS/WBRT (gefitinib/erlotinib + SRS/WBRT) as well as EGFR-TKIs + anti-VEGFR (erlotinib + bevacizumab) are preferred therapies for prolonging PFS. EGFR-TKIs + SRS/WBRT are likely to provide the greatest OS benefit. EGFR mutations had a significant association with OS but not PFS in NSCLC patients, after considering multiple factors. In the SEER analysis, the 6-month, 1-year, 3-year survival rates were 59.8%, 41.3%, and 5.6%, respectively. After multivariable Cox analyses, race and origin, and year of diagnosis were independent predictors for the OS of the brain metastatic populations. Patients with cystic, mucinous and serous tumours and white patients had longer OS than those with other histology and races.

Recurrence, metastasis and resistance are well-known problems in cancer treatment [29]. For EGFR-mutant NSCLC patients, EGFR-TKIs are the standard first-line regimens; however, therapeutic challenges remain in subpopulations, notably those with brain metastases, who have a poor prognosis [30]. Osimertinib was significantly superior to other treatments for CNS metastases in NSCLC patients with EGFR mutations, especially among the T790M-positive patients [31]. As a substrate of the permeability glycoprotein, osimertinib has shown greater penetration of the blood-brain barrier than gefitinib, rociletinib, or afatinib, and it is also more widely distributed in the primary brain, which accounts for the satisfying outcomes observed to date in brain-metastatic EGFR-mutant NSCLC patients [18]. Pooled data from clinical trials showed that the objective response rate (ORR) of T790M-positive patients with CNS metastasis was 54% with a higher response duration and a good safety profile [32], and the CNS ORR was 70% in the AURA17 trial of osimertinib [33]. If patients acquired T797S resistance, the response to osimertinib seemed not so dramatic. Evidence showed almost 25% patients had MET amplification with positive T797S, at that time, these patients would get more than 20% ORR from combined therapy with MET inhibitor plus EGFR-TKIs (i.e. osimertinib plus tivantinib or erlotinib plus tivantinib) [17, 34]. Prior RT may expand the efficacy of osimertinib in brain-metastatic patients based on evidence showing that RT increases the penetration of EGFR-TKI through the blood-brain barrier. Here, the authors suggested that EGFR-TKIs + SRS/WBRT was also a favourable treatment option [35, 36], however, SRS and WBRT presented by included trials but were grouped as single SRS/WBRT network nodes in our study, which could be a potential source of heterogeneity and inconsistency, and possible weak transitivity though no significant inconsistency was observed. In advanced/metastatic EGFR-mutant NSCLC, gefitinib, erlotinib and afatinib have been previously confirmed to have superior efficacy and fewer adverse events compared to platinum-based chemotherapy [25, 37]. Afatinib was statistically superior to gefitinib/erlotinib, but there was no significant trend to improve the PFS and OS of intended populations. WBRT is widely used before for NSCLC patients with multiple brain metastases, while elevated EGFR expression is an important cause of resistance to RT. In these situations, the use of TKIs (e.g., EGFR-TKIs) causes the lesions to be more radiosensitive [38]. Dramatically, detailed sub-types of EGFR mutations, history of definitive treatment, RT and other therapies might be varied across included studies, which remind the practioners to take these biases into consideration before real clinical practice. In summary, it is believed to be a comprehensive study regarding therapies for brain-metastatic, EGFR-mutant NSCLC patients, and it may provide the highest level of evidence for both physicians and patients, also influence the national/international medical guidelines.

Reviewing the results of individual patient data, white patients, especially non-Hispanic white patients, often have more access to health care, which causes accurate diagnosis and early treatment; thus, they have longer survival times [39]. Similarly, an early year of diagnosis also leads to proper prevention and treatment, hence prolonging the OS. Cystic, mucinous and serous neoplasms were associated with significantly longer OS in the SEER analysis (log-rank P < 0.001), regardless of treatment; however, it was possible that the “true” histological classification could be confounded by the diagnostic method or the reporting scheme of the guidelines, not to mention heterogeneous classification. Socioeconomic status was found to be associated with the substantially improved survival of brain-metastatic NSCLC patients, but this study cannot provide adequate evidence [40]. The survival times of populations was not significantly different according to surgery status (performed or not), suggesting that only the treatment was insufficient to explain the survival differences [41, 34]. At that time, biomarkers test for targeted therapies or multiple disciplinary team (MDT) strategies with other adjuvant therapeutics were sensible. Currently, Graded Prognostic Assessment (GPA) using “age”, “Karnofsky performance score”, “extracranial metastases”, “brain metastatic lesions” as four items is a well-accepted index for brain-metastatic NSCLC patients [42]. A more accurate and obtainable diagnosis-specific tool will assist in discerning the appropriate treatments.

Current relevant national and international guidelines are mostly based on the results of single RCTs and standard pooled studies involving pairwise comparisons of 2 or 3 regimens. Conventional pairwise pooled studies of direct comparisons are inevitably limited by the relatively small number of studies assessing a particular pair of treatments. We note that the current study is the first to address the efficacy of therapeutics for EGFR-mutant NSCLC with brain metastasis with state-of-the art Bayesian methods. The conclusions are strengthened by these merits: first, we identified two regimens that were potentially superior to others on the basis of RCT data, which makes the interpretation to clinical work with more confidence. Second, the current study is based on multivariable, time-varying hazard ratios that assumed proportional hazards, examined the relative treatment efficacy based on parameters of survival plots (shape and scale), and considered the influence of time [34]. Network studies regarding relative risk or odds ratio do not have these strengths. Third, an extensive search assured that all eligible studies were included, increasing our ability to estimate comparisons across the network. The combination of direct and indirect evidence, which was not restricted to head-to-head comparisons, contributes to the extensive applicability of the conclusion and provides more clinically relevant information. Fourth, a prognostic model with good concordance for brain-metastatic NSCLC patients was initially updated to provide more guidance and reference regarding the assessment of prognosis.

### Limitations

There are still several limitations needed to be pinpointed. A few important variables, such as EGFR mutation subtypes, the status of extra-cranial disease control, the use of salvage systematic therapies, dose of RT and health-related quality of life, have been inconsistently reported [43]. Moreover, this study mainly incorporated CNS asymptomatic or stable patients, therefore, the conclusion should be interpreted with caution for patients with symptomatic brain disease. It should also be acknowledged that patients with severe neurological disorders or leptomeningeal metastases might not mostly get benefits from the discussed treatments and their life expectancy was poor. Though there was a comprehensive search, regimens (10 classes) and patients (1710 patients) lacking power were selected for the evidence base, leading to a sparse network, with some treatments (i.e., deferring SRS/WBRT, EGFR-TKIs + MET-TKIs) only investigated by a single trial. In fact, comparisons at the individual-treatment level and treatment-class level would be inadvertently biased, as not all therapies could be included in both network scopes. In the current study, treatment-class level analyses were purposefully performed based on the available data, trying to comprehensively embody the available data. Finally, in the prognostic model, a number of important characteristics of individuals could not be successfully obtained, such as targeted molecules, RT or chemotherapy history. Thus, a more standardized and precise model is warranted in the future.

## CONCLUSIONS

The third-generation EGFR-TKI and EGFR-TKIs + SRS/WBRT have well-known superiority for EGFR-mutant NSCLC patients with brain metastases, with acceptable safety profiles. Benefits from EGFR-TKIs + anti-VEGFR still need to be validated and expanded. EGFR-mutant NSCLC patients have longer OS compared to wild-type NSCLC patients. Surgery without further adjuvant therapies status has no association with the survival of brain-metastatic NSCLC patients; however, it can provide accurate diagnoses and relieve symptoms. Preparations of molecular analyses for targeted therapy with access to appropriate supportive care are optimal. The findings challenge the efficacy of first-/second-generation EGFR-TKIs again and address the current landscape of the use of third-generation TKIs for the treatment of EGFR-mutant advanced/metastatic NSCLC. Based on this sparse network, these conclusions need to be reinforced and updated with novel therapies emerging in the future.

## MATERIALS AND METHODS

This Bayesian study was reported according to the Preferred Reporting Items for Systematic Reviews and Meta-Analyses (PRISMA) extension statement for network meta-analysis for health care (Appendix Table A1) [44]. No conflict of interest was involved (PROSPERO registration information: CRD42019127525).

### Search strategy

PubMed, EMBASE, Cochrane Library, Web of Science and ClinicalTrials.gov were rigorously searched from inception to Nov 30 2019 without language restrictions for randomized controlled trials (RCTs) regarding treatment options for EGFR-mutant NSCLC patients with brain metastases. The search details can be found in Appendix Doc 1. We manually searched the reference lists of the retrieved studies and grey literature for additional records.

### Selection criteria

Eligible studies had to meet:

1. Populations: Adult (≥ 18 y) histologically or cytologically confirmed NSCLC patients with sensitizing EGFR mutations and asymptomatic or neurologically stable brain metastases. Eligible participants had an Eastern Cooperative Oncology Group (ECOG) performance score of 0 or 1, a life expectancy of at least 3 months and certain levels of organ function (bone marrow, liver, kidney function etc.). There were no restrictions regarding other characteristics.
2. Interventions and comparisons: Reasonable regimens (including surgery, pharmaceutical intervention, and RT).
3. Outcome: At least PFS or overall survival (OS) had to be reported. Adverse effects might also be reported.
4. Study design: phase II/III RCTs or randomized trial lasting at least one year.

Only ASCO, ESMO, IASLC, SNO conference abstracts were considered. Studies involving patients with confirmed metastases in the spinal cord or leptomeningeal, who had less than 3 months life expectancy were excluded [45]. Case reports, basic research, reviews, and meta-analyses were also excluded. More information is provided in Appendix Doc 1.

### Data extraction and quality assessment

Two authors (Zhao-B. H. and Wang-Y.) independently extracted useful information from primary studies. A final decision was reached after a discussion with a third author (Ma-W.B.) in case of any discrepancies. We contacted the primary authors for additional information if the necessary data could not be extracted or obtained by other methods.

The quality of each trial was assessed with the modified version of the Cochrane Risk of Bias tool [46]. More descriptions of the data extraction and quality assessments are provided in Appendix Doc 1.

### Classification of treatment arms

To organize the existing options tested in clinical trials into clinical meaningful arms, we used general classes criteria shown in Appendix Doc 1. Related SRS and WBRT were combined into SRS/WBRT for insufficient data.

The prespecified primary outcome was PFS, and the secondary outcome was OS in this study. We also analysed adverse effects during therapies to address the potential safety concerns.

### Statistical analyses

The Bayesian network meta-analysis (NMA) was performed with a random effects model to estimate the HR and 95% credible interval (95% Crl) for PFS and OS between trial arms [47]. In studies with directly unavailable HR, we extracted and estimated the HR and corresponding standard errors from the Kaplan–Meier curves, if available, with the methods described by Tierney et al [48]. In the case of multi-arm trials (trials with three or more interventions), adjustments were made to preserve randomization and correlation within multi-arm trials by converting log-HRs to log-hazards [49, 50]. Markov Chain Monte Carlo (MCMC) methods were used to obtain the data, and we evaluated the inconsistency of the model by the edge-splitting method based on all direct and indirect evidence [51]. Relative treatment rankings were displayed graphically with rankograms [52].

In the Bayesian context, statistical significance was established when the 95% Crl did not contain 1. Calculations were performed in R version 3.5.3 (www.r-project.org) using the public gemtc and jag packages. The parameters and details of those methods can be found in Appendix Doc 1.

### Prognostic model of individual patient data

Initially, the authors detected the prognosis (PFS and OS here) of NSCLC patients by EGFR mutations through the online Kaplan-Meier plotter (http://kmplot.com/analysis) [53]. Survival curves of the endpoints were plotted online for the total and subgroup populations, and the log-rank P value was calculated automatically by the online tool.

To allow for more flexible modelling, the authors further retrieved individual patient survival data from the National Cancer Institute Surveillance, Epidemiology, and End Results registries (SEER) database using SEER*Stat software from Jan 1, 2000, to Dec 1, 2015 [54]. Populations were restricted to those with brain-metastatic NSCLC (M1). The data included age, sex, race, race and origin (Hispanic, non-Hispanic), histology (acinar cell neoplasms, adenomas and adenocarcinomas, complex epithelial neoplasms, complex mixed and serous neoplasms, cystic mucinous and serous neoplasms, epithelial neoplasms and squamous cell neoplasms), year of diagnosis, stage_T, stage_N, surgery status (both primary and metastatic lesions), tumour size (<100, 100—199, ≥200 0.1 mm), survival status and survival time (months). Non-Hispanic was further classified as non-Hispanic Asian or Pacific islander, non-Hispanic black, and non-Hispanic white; to obtain the newest data, the year of diagnosis was classified as 2010-2013 and 2014-2015. Histologic groups were classified using the International Classification of Disease (ICD) for Oncology, Third Edition [55]; stage_T and stage_N were categorized based on the American Joint Committee on Cancer (AJCC) TNM classification 8^th^ Edition [56].

The impacts of several clinical factors on the 6-month, 1-year and 3-year OS rates of patients were quantitatively summarized in the nomogram by a multivariable Cox proportional hazard model; the performance of the nomogram was assessed by the concordance index (C-index) and evaluated by comparing the nomogram-predicted versus real estimates of survival probability visually on a calibration curve. Survival curves were generated using the Kaplan-Meier (K-M) method and compared using the log-rank test. A log-rank P value < 0.05 indicated significant survival difference. The authors used R with the ggplot, ggsurvplot, and SEER public packages to perform the statistical analyses. The details are presented in Appendix Doc 1.

### Ethics approval

This is a secondary study based on primary RCTs. Participants enrolled in the primary RCTs had reported approval and consent on ethics

## Supplementary Material

Supplementary Methods

Supplementary Figures

Supplementary Table 1, Appendix Table A1

Supplementary Tables 2 and 3
