# Supplementary material for: Efficacy and safety of therapies for EGFR-mutant non-small cell lung cancer with brain metastasis: an evidence-based Bayesian network pooled study of multivariable survival analyses
Source: Aging (Albany NY). 2020 Jul 15;12(14):14244–70. doi: 10.18632/aging.103455 (PMC7425486; doi:10.18632/aging.103455)
Supplement: Supplementary Methods [file aging-12-103455-s002..pdf]

SUPPLEMENTARY METHODS

This document summarizes clear and detailed methods in the study

Search strategy

	PubMed/Web of Science Search Strategy
**1 non-small cell lung cancer**	("Carcinoma, non-small cell lung"[Mesh] OR " carcinomas, non-small cell lung" OR "lung carcinoma, non-small cell" OR " lung carcinomas, non-small cell " OR "non-small cell lung carcinomas" OR "nonsmall cell lung cancer" OR "non-small-cell lung carcinoma" OR "non small cell lung carcinoma" OR "carcinoma, non-small cell lung " OR "non-small cell lung cancer")
**2 EGFR mutant**	("EGFR" OR "EGFR-mutant" OR "EGFR mutations" OR "egfr" OR "aerobic capacity" OR " epidermal growth factor receptor" OR "EGFR-mutant patients" OR "patients with EGFR mutations")
**3 brain metastasis**	("brain metastasis" OR "CNS metastasis" OR "advanced cancer" OR "advanced carcinoma" OR "multiple-metastasis" OR "multiple metastasis lesions")
**4 RCT**	(randomized controlled trial[pt] OR controlled clinical trial[pt] OR randomized[tiab] OR placebo[tiab] OR clinical trials as topic[mesh:noexp] OR randomly[tiab] OR trial[ti])
**5**	(animals[mh] NOT humans [mh])
**6**	1 AND 2 AND 3 AND 4
**7**	6 NOT 5

Selection criteria

Eligible studies had to meet the following issues:

(1) Populations: Adult (≥ 18 y) histologically or cytologically confirmed NSCLC patients with sensitizing EGFR mutations and with asymptomatic or neurologically stable brain metastasis. Eligible participants had an Eastern Cooperative Oncology Group (ECOG) performance score of 0 or 1; brain metastatic lesions could be measurable according to Response Evaluation Criteria in Solid Tumors (RECIST), version 1.1; life expectancy of at least 3 months and certain organ function (bone marrow, liver, kidney function etc.) according to the National Cancer Institute Common Terminology Criteria for Adverse Events (NCI-CTCAE) version 4.0. There were no restrictions on other characteristics.
(2) Interventions and comparisons: Reasonable interventions and comparisons (including surgery, pharmaceutical intervention, radiotherapy etc.).
(3) Outcome: At least reported PFS and overall survival. Adverse effects of investigated therapies might also be reported.
(4) Study design: RCTs or in randomized clinical form lasting at least one year.

Conference abstracts only from American Society of Clinical Oncology (ASCO), European Society for Medical Oncology (ESMO), the International Association for the Study of Lung Cancer (IASLC), the Society for Neuro-oncology (SNO) were considered, the eligible records should have total or part essential information. Studies involved patients with confirmed metastases in the spinal cord or leptomeningeal, who had less than 3 months life expectancy were excluded. Case reports, fundamental researches, reviews, meta-analyses were also excluded.

Data extraction and quality assessment

Two authors independently extracted the following information from the eligible studies: name of the first author, nation, baseline characteristics of participants (sex, mean age), strategies of intervention and comparison in each arm, hazard ratios (HRs) of the intended outcomes in the fully adjusted model, study duration and study type. In the case of any discrepancies, a final decision was reached after a discussion with a third author. The acquired information was partly entered into a standardized table. We contacted the primary author for additional information if the data could not be extracted or obtained by other methods.

The quality of each trial was assessed with the modified version of the Cochrane Risk of Bias tool regarding random sequence generation, allocation concealment, blinding of participants and personnel, blinding of outcome assessment, incomplete outcome data, selective reporting, and other sources of bias. The quality of each study was categorized as high, low, or unclear; low and unclear quality meant that there was a high risk of bias [1].

Intervention and outcome classification

Current eligible studies incorporated 10 medication classes:

Medication class 1. platinum-based chemotherapy: Cisplatin + pemetrexed, Cisplatin + gemcitabine, Pemetrexed + carboplatin or cisplatin,

Medication class 2. first generation EGFR-TKI: Gefitinib, Erlotinib, Icotinib,

Medication class 3. second generation EGFR-TKI: Afatinib,

Medication class 4. third generation EGFR-TKI: Osimertinib,

Medication class 5. EGFR-TKIs + platinum-based chemotherapy: Gefitinib + cisplatin and pemetrexed

Medication class 6. EGFR-TKIs + SRS/WBRT: Gefitinib/erlotinib + stereotactic radiosurgery (SRS)/whole brain radiotherapy (WBRT)

Medication class 7. deferring SRS/WBRT

Medication class 8. WBRT

Medication class 9. EGFR-TKIs + MET-TKIs: Erlotinib + tivantinib

Medication class 10. EGFR-TKIs + anti-VEGFR: Erlotinib + bevacizumab

EGFR mutations were evaluated in biopsy specimens or bronchoalveolar lavage fluid from each patient at a commercial central laboratory. The prespecified primary outcome was PFS (defined as the time from randomization to the progression of primary/metastatic disease, withdrawal, or death from any cause), and the secondary outcome was overall survival (OS), which was determined by primary investigators according to RECIST version 1.1 in the Bayesian study. We also analysed adverse effects during therapies to address the potential safety concerns.

Statistical analyses

The Bayesian network meta-analysis (NMA) was performed with a random effects model to estimate the HR and 95% credible interval (95% Crl) for PFS and OS between trial arms [2]. In studies in which HR was not provided directly, we extracted and estimated the HR and corresponding standard errors from the Kaplan–Meier curves, if available, with the methods described by Tierney et al[3]. In the case of multi-arm trials (trials with three or more interventions), adjustments were made to preserve randomization and correlation by converting log-HRs to log-hazards [4, 5].

Markov Chain Monte Carlo (MCMC) methods were used with the obtained data, and the fit of the random effects model was assessed by the deviance information criteria (DIC). A three-chain model with non-informative priors was run with an adaptation phase of 10000 iterations followed by 100000 model iterations. The thin ratio was set to 10. Non-convergence was assessed by the Gelman-Rubin statistic. Relative treatment rankings (probability for each treatment to be the most effective (first best regime), the second best, the third best and so on) were displayed graphically with rankograms [6], which indicated the probable best and worst therapies. We evaluated inconsistency by the edge-splitting method, an approach estimating relative treatment effects based on direct evidence (pairwise comparisons between treatment nodes) and indirect evidence (relative treatment effects estimated using indirect evidence) [7]. In case of significant inconsistency, the authors investigated the distributions of clinical and methodological variables that might be potential sources of either heterogeneity or inconsistency in every comparison-specific group of trials. We used visual inspection of the forest plots to investigate the possibility of statistical heterogeneity. This inspection was supplemented with I^2 statistics, which provided an evaluation of the percentage of variability due to heterogeneity rather than a sampling error [8]. An I^2 statistic > 50% was regarded as indicating significant heterogeneity. Trace, density and comsol estimations/plots were used to inspect the uncertainty of the MCMC model [8]. In the Bayesian context, statistical significance was established when the 95% Crl did not contain 1. Calculations were performed in R version 3.5.3 (https://www.r-project.org) using the gemtc [9] and jag packages.

Clinical model of individual patient data

To allow for more flexible modelling, the authors reconstructed individual patient survival data from the National Cancer Institute Surveillance, Epidemiology, and End Results registries (SEER) database using SEER*Stat software from Jan 1, 2000, to Dec 1, 2015 [10]. The population was restricted to brain-metastatic NSCLC (M1) patients. The data included age (<60, 60-69, 70-79, ≥80 y), sex (female, male), race (black, white, other), origin (Hispanic, non-Hispanic), histology (acinar cell neoplasms, adenomas and adenocarcinomas, complex epithelial neoplasms, complex mixed and serous neoplasms, cystic mucinous and serous neoplasms, epithelial neoplasms and squamous cell neoplasms), year of diagnosis (2010-2013, 2014-2015), stage_T (T0, T1, T2, T3, T4, T5, TX), stage_N (N0, N1, N2, N3, NX), surgery status (performed, not performed), tumour size (<100, 10—199, ≥200 0.1 mm), survival status (alive, dead) and survival time (months). Non-Hispanic was further classified as non-Hispanic Asian or Pacific islander, non-Hispanic black, and non-Hispanic white [11]; to obtain the newest data, the year of diagnosis was classified as 2010-2013 and 2014-2015. Histologic groups were classified using the International Classification of Disease (ICD) for Oncology, Third Edition [12]; stage_T and stage_N were categorized based on the American Joint Committee on Cancer (AJCC) TNM classification 8th Edition [13].

The influence of several clinical factors on the 6-month, 1-year and 3-year OS rates of patients was summarized

in the nomogram by a multivariable Cox proportional hazard model; the performance of the nomogram was assessed by the concordance index (C-index) and evaluated by comparing nomogram-predicted versus real estimates of survival probability visually on a calibration curve. Survival curves were generated using the Kaplan-Meier method and compared using the log-rank test. A P value < 0.05 was regarded as statistically significant. The authors used R version 3.5.3 with the ggplot [14], ggsurvplot, and SEER packages[15 to perform the statistical analyses.

Supplementary References

1. Higgins JP, Altman DG, Gøtzsche PC, Jüni P, Moher D, Oxman AD, Savovic J, Schulz KF, Weeks L, Sterne JA, and Cochrane Bias Methods Group, and Cochrane Statistical Methods Group. The cochrane collaboration's tool for assessing risk of bias in randomised trials. BMJ. 2011; 343:d5928. https://doi.org/10.1136/bmj.d5928 PMID:22008217

2. Lu G, Ades AE. Combination of direct and indirect evidence in mixed treatment comparisons. Stat Med. 2004; 23:3105–24. https://doi.org/10.1002/sim.1875 PMID:15449338

3. Tierney JF, Stewart LA, Ghersi D, Burdett S, Sydes MR. Practical methods for incorporating summary time-to-event data into meta-analysis. Trials. 2007; 8:16. https://doi.org/10.1186/1745-6215-8-16 PMID:17555582

4. Woods BS, Hawkins N, Scott DA. Network meta-analysis on the log-hazard scale, combining count and hazard ratio statistics accounting for multi-arm trials: a tutorial. BMC Med Res Methodol. 2010; 10:54. https://doi.org/10.1186/1471-2288-10-54 PMID:20537177

5. Salanti G, Higgins JP, Ades AE, Ioannidis JP. Evaluation of networks of randomized trials. Stat Methods Med Res. 2008; 17:279–301. https://doi.org/10.1177/0962280207080643 PMID:17925316

6. Salanti G, Ades AE, Ioannidis JP. Graphical methods and numerical summaries for presenting results from multiple-treatment meta-analysis: an overview and tutorial. J Clin Epidemiol. 2011; 64:163–71. https://doi.org/10.1016/j.jclinepi.2010.03.016 PMID:20688472

7. Dias S, Welton NJ, Caldwell DM, Ades AE. Checking consistency in mixed treatment comparison meta-analysis. Stat Med. 2010; 29:932–44. https://doi.org/10.1002/sim.3767 PMID:20213715

8. Higgins JP, Thompson SG, Deeks JJ, Altman DG. Measuring inconsistency in meta-analyses. BMJ. 2003; 327:557–60. https://doi.org/10.1136/bmj.327.7414.557 PMID:12958120

9. van Valkenhoef G, Kuiper J. gemtc. Network Meta-Analysis Using Bayesian Methods. R package version 0.8–2.

10. National Cancer Institute Surveillance Epidemiology and End Results Program. Overview of the SEER program. http://seer.cancer.gov/about/overview.html. Accessed January 31, 2018.

11. NAACCR Race and Ethnicity Work Group. NAACCR guideline for enhancing Hispanic/Latino identification: revised NAACCR Hispanic/Latino identification algorithm [NHIA v2.2.1]. https://www.naaccr.org/wp-content/uploads/2016/11/NHIA-v2.2.1.pdf. Revised September 12, 2011. Accessed January 31, 2018.

12. Fritz APC, Jack A, Shanmugaratnam K, Sobin L, Perkin DM, Whelan S, eds. International Classification of Diseases for Oncology. 3rd ed. Geneva, Switzerland: World Health Organization; 2000.

13. Egner JR. AJCC Cancer Staging Manual. JAMA. 2010;304:1726–1727. https://doi.org/10.1001/jama.2010.1525

14. Wickham H. ggplot2: Elegant Graphics for Data Analysis. New York: Springer Science+Business Media, LLC; 2009.

15. Luo J. Reading and writing SEER*STAT data files. Package 'SEER2R.' https://cran.r-project.org/web/packages/SEER2R/index.html. Published February 19, 2015. Accessed January 31, 2018.
