# Supplementary material for: Efficacy and safety of therapies for EGFR-mutant non-small cell lung cancer with brain metastasis: an evidence-based Bayesian network pooled study of multivariable survival analyses
Source: Aging (Albany NY). 2020 Jul 15;12(14):14244–70. doi: 10.18632/aging.103455 (PMC7425486; doi:10.18632/aging.103455)
Supplement: Supplementary Figures [file aging-12-103455-s004..pdf]

SUPPLEMENTARY FIGURES

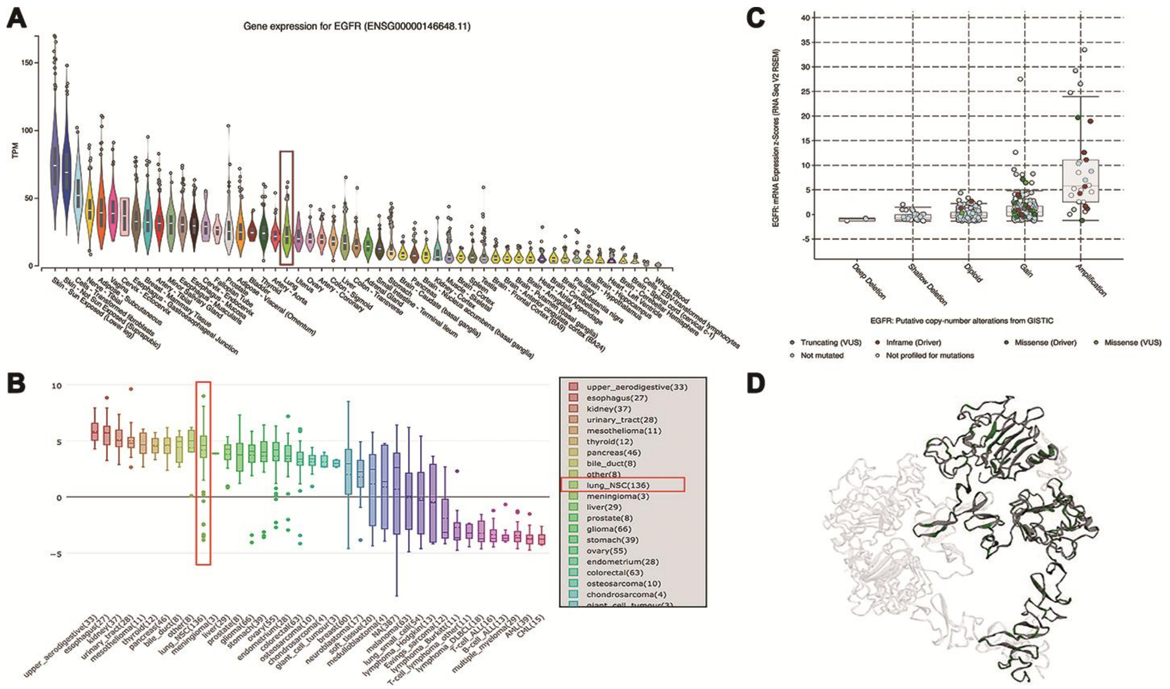

Supplementary Figure 1. The landscape of the EGFR gene and mutations. (**A**) Gene expression for EGFR in normal tissues (CCLE analysis **https://portals.broadinstitute.org/ccle**), (**B**) gene expression for EGFR in cancer cell lines (CCLE analysis), (**C**) EGFR putative copy-number alternations from GISTIC (online source from **http://www.cbioportal.org/**), (**D**) 3D structure of the EGFR protein. EGFR mutations were commonly detected in normal lung tissues, ranking 21st in the 53 reported organ/tissue cells and 10th in the 37 pan-cancer types in NSCLC (Figure A1A, A1B). EGFR putative copy-number alternations from GISTIC based on 584 patients (containing 586 samples, from *CBioPortal*) harboured deep deletion, shallow deletion, diploid, gain and amplification mutations, among which amplification was the most commonly detected in the mRNA (A1C). The 3D structure of the EGFR protein is depicted in Figure A1D in dark green

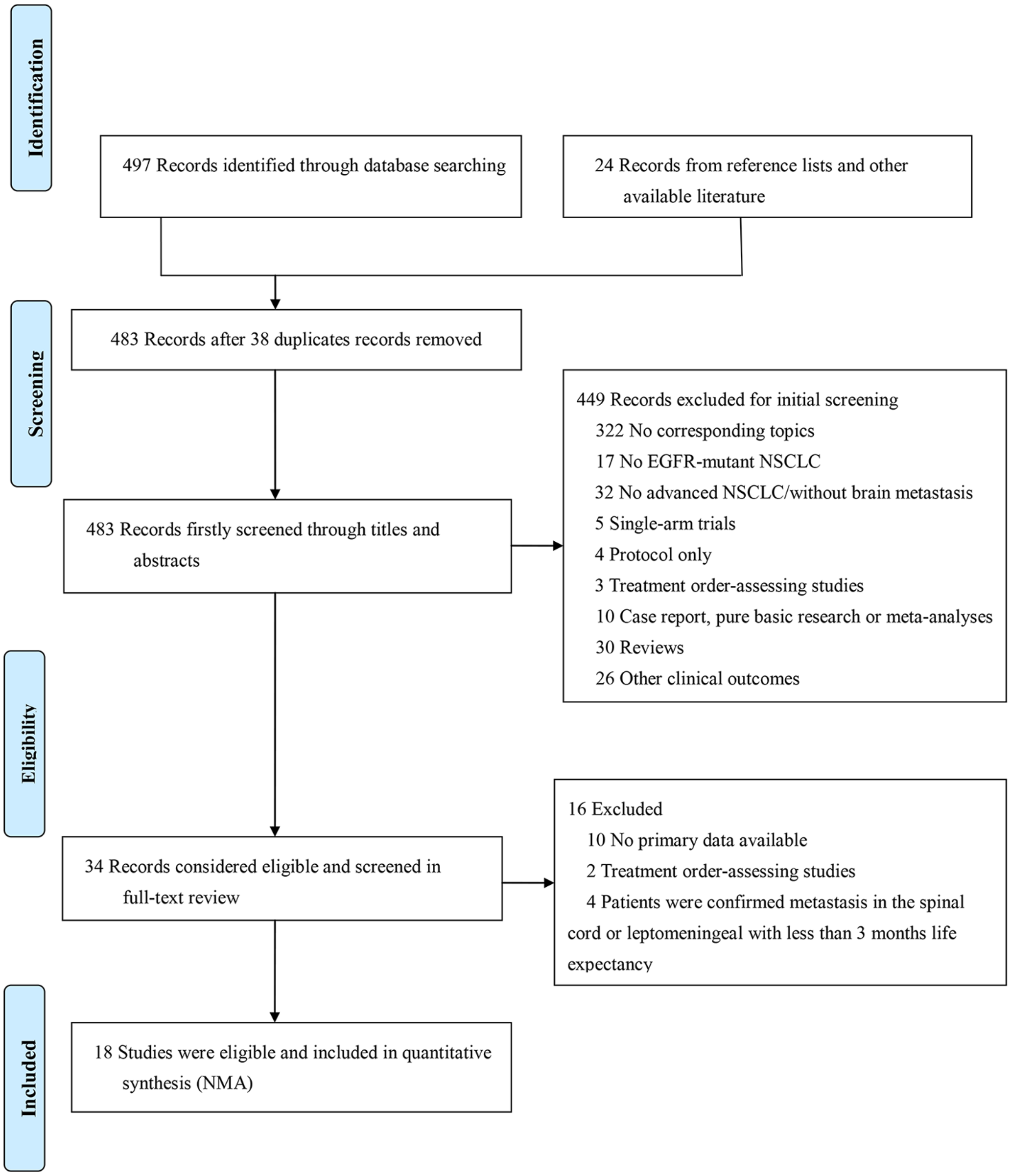

Supplementary Figure 2. Study flow chart.

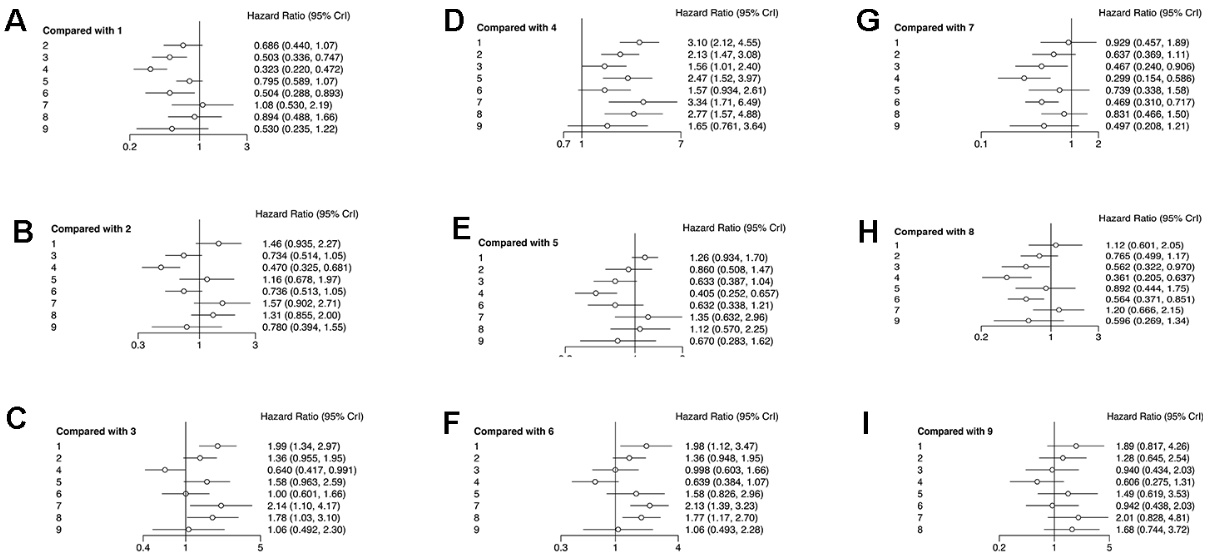

Forrest plots of multivariable interventions for PFS

Interventions note:

1: Platinum-based chemotherapy
2: First generation EGFR-TKI
3: Second generation EGFR-TKI
4: Third generation EGFR-TKI
5: EGFR-TKIs + platinum-based chemotherapy
6: EGFR-TKIs + SRS/WBRT
7: Deferring SRS/WBRT
8: WBRT
9. EGFR-TKIs + anti-VEGFR

Supplementary Figure 3. Forrest plots for network comparisons of multiple-treatments on PFS

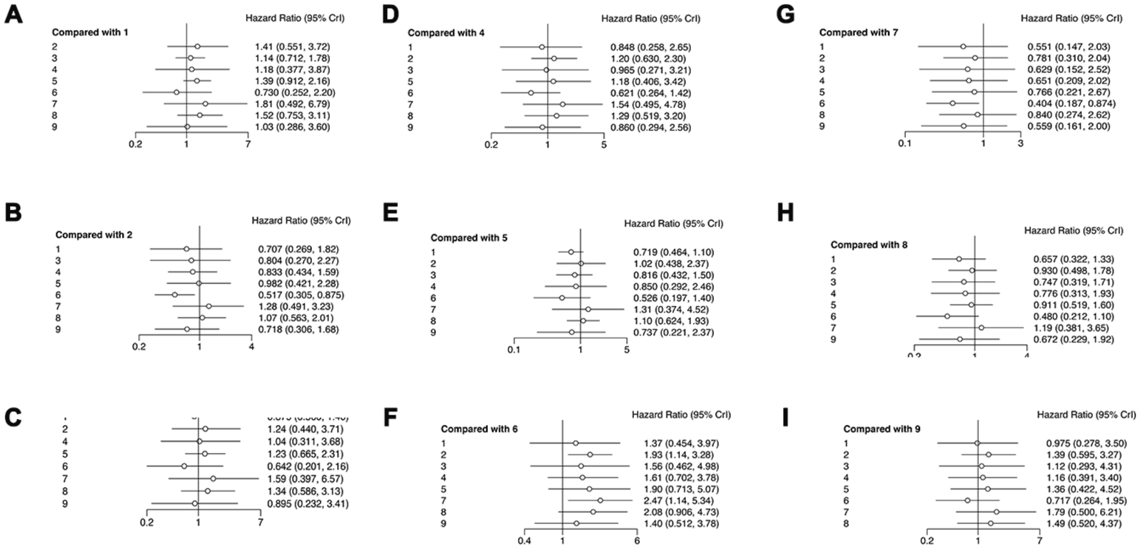

Forrest plots of multivariable interventions for OS

Interventions note

1.Platinum-based chemotherapy
2 First generation EGFR-TKI
3. Second generation EGFR-TKI
4. Third generation EGFR-TKI
5. EGFR-TKIs + platinum-based chemotherapy
6. EGFR-TKIs + SRS/WBRT
7. Deferring SRS/WBRT
8.WBRT
9. EGFR-TKIs + MET-TKIs

Supplementary Figure 4. Forrest plots for network comparisons of multiple-treatments on OS

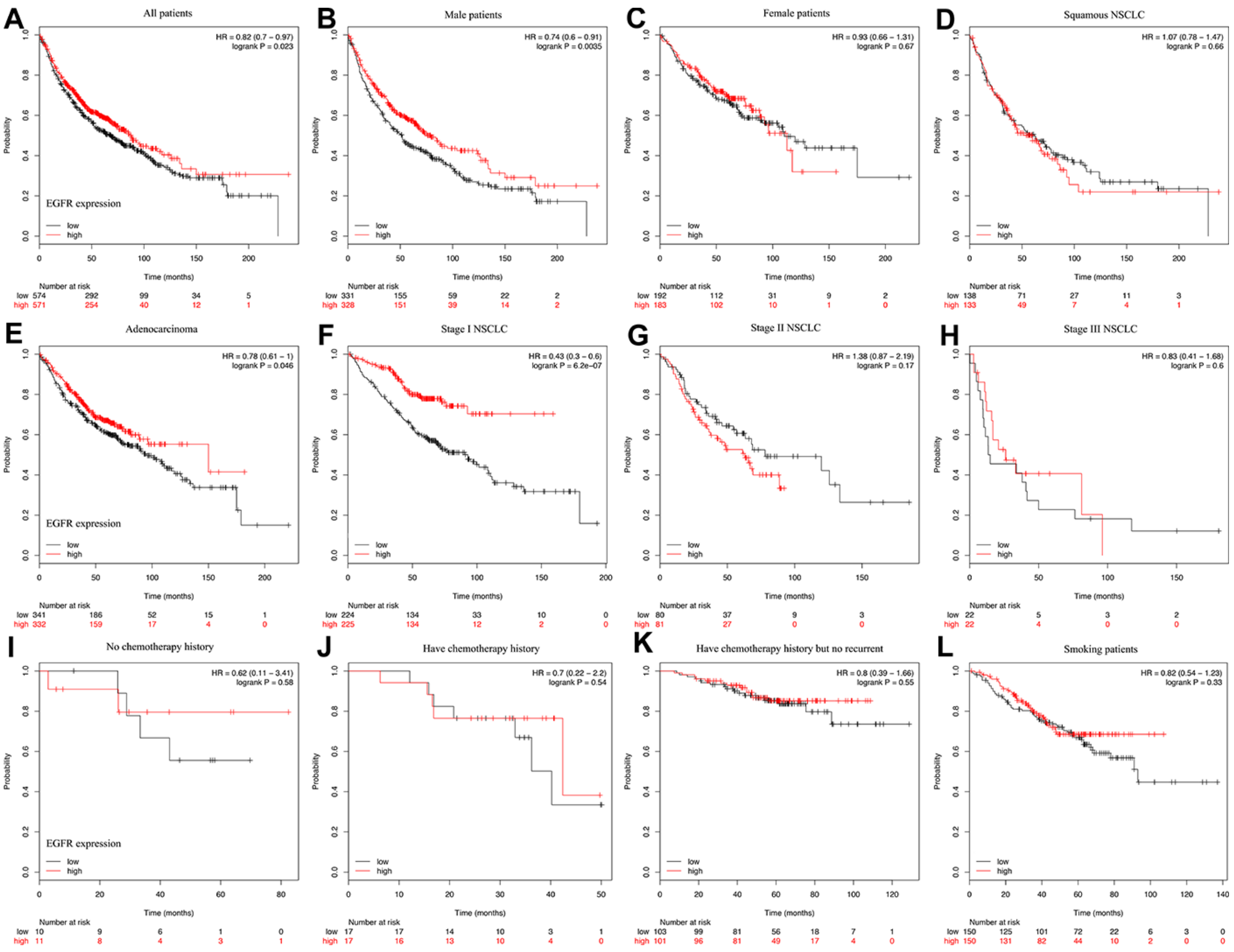

Supplementary Figure 5. Kaplan-Meier plots by EGFR mutations for OS

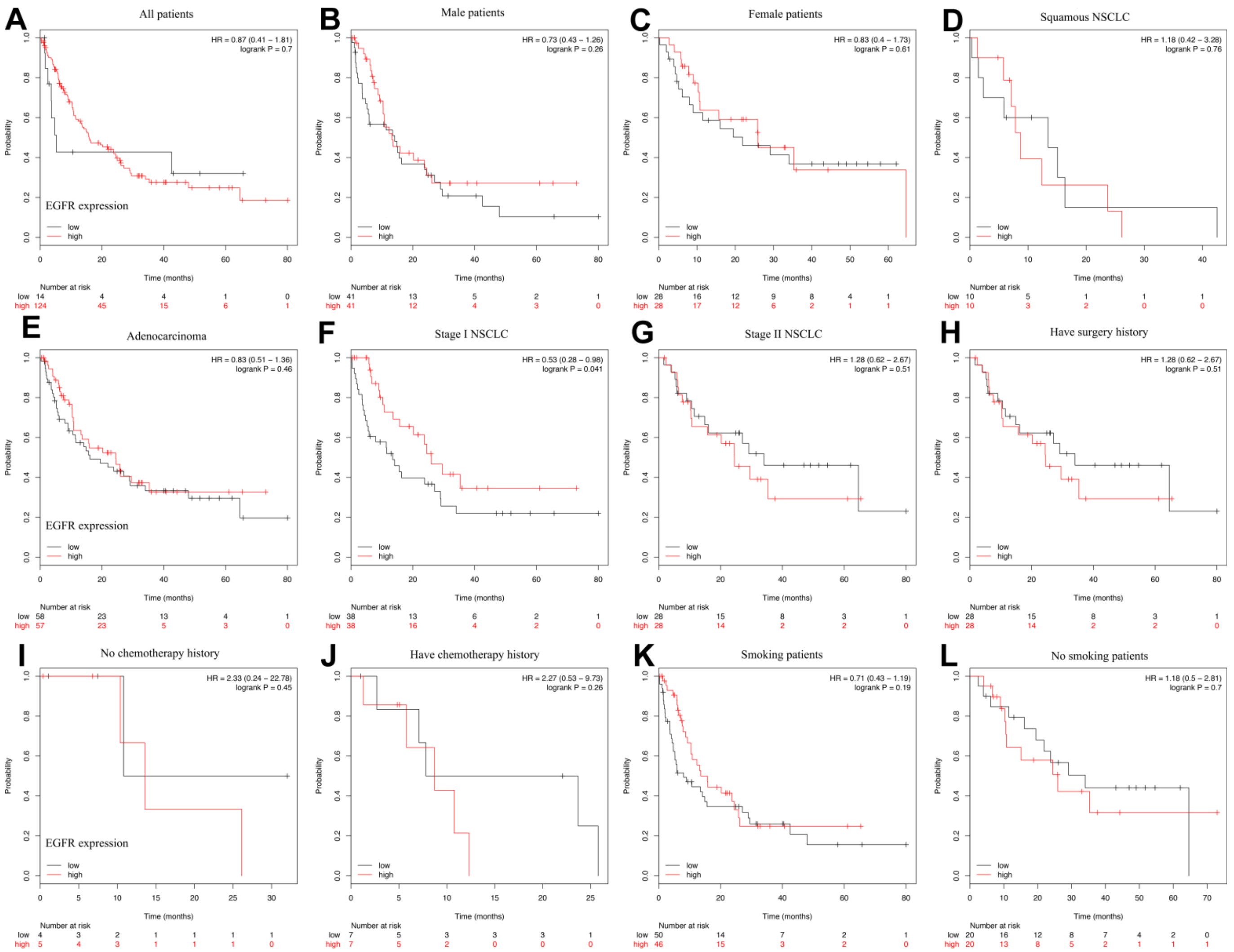

Supplementary Figure 6. Kaplan-Meier plots by EGFR mutations for PFS.
