# Supplementary material for: Efficacy and safety of therapies for EGFR-mutant non-small cell lung cancer with brain metastasis: an evidence-based Bayesian network pooled study of multivariable survival analyses
Source: Aging (Albany NY). 2020 Jul 15;12(14):14244–70. doi: 10.18632/aging.103455 (PMC7425486; doi:10.18632/aging.103455)
Supplement: Supplementary Tables 2 and 3 [file aging-12-103455-s003..pdf]

SUPPLEMENTARY TABLES

Supplementary Table 2. Methodology quality of the included studies.

	Random sequence generation *(Selection bias)*	Allocation concealment *(Selection bias)*	Blinding of participants and personnel *(Performance bias)*	Blinding of outcome assessment *(Detection bias)*	Incomplete outcome data *(Attrition bias)*	Selective reporting *(Reporting bias)*	Other sources of bias‡
**Sequist, 2013**	+	–	–*	–*	+	+	+
**Wu, 2014**	+	–	–*	–*	+	+	+
**Scagliotti, 2015**	+	+	+	+	+	–	–
**Soria, 2015**	+	+	+	–	–	+	+
**Magnuson, 2016**	+	–	–	–	+	+	–
**Park, 2016**	+	+	+	+	+	+	+
**Schuler, 2016**	+	–	–	–	+	+	+
**Fan, 2017**	–	–	–	–	+	+	–
**Mok, 2017**	+	+	–	–	–	+	–
**Mok (2), 2017**	+	–	–*	–*	+	+	+
**Yang, 2017**	+	+	–*	–*	+	+	+
**Zhu, 2017**	+	–	–	–	+	+	–
**Reungwetwattana, 2018**	+	+	+	+	+	–	–
**Soria, 2018**	+	+	+	+	+	+	–
**Wu, 2018**	+	–	–*	–*	–	+	+
**Yang, 2018†**	+	+	–	–	–	+	–
**Saito, 2019**	+	+	–	–	+	+	–
**Ramalingam, 2019**	+	+	+	+	+	+	+

+ = Low risk of bias – =High risk of bias

*Open label controlled trials.

† Abstract including useful and essential data presented in IASLC conference

‡ can not give precise judgements on other quality-assessment items.

Supplementary Table 3. Summarized Cox hazard ratio-regression outcomes of comparisons according to SEER database.

Groups	Number of individuals	Effective estimate (HR)	P-value
**Age (y)**	1173		
**< 60**	59	1 (Ref)	
**60-69**	197	1.17 (0.63-1.70)	0.879
**70-79**	416	1.38 (0.79-1.97)	0.350
**≥ 80**	501	1.21 (0.66-1.76)	0.762
**Sex**	1173		
**Female**	592	1 (Ref)	
**Male**	581	1.01 (0.79-1.23)	0.881
**Race**	1173		
**Black**	112	1 (Ref)	
**White**	1047	0.75 (0.45-1.04)	0.076
**Other***	14	1.64 (0.48-2.80)	0.743
**Race and origin**	1173		
**Hispanic**	41	1 (Ref)	
**Non-Hispanic†**	1132	0.64 (0.31-0.97)	0.037
**Histology**	1173		
**Acinar cell neoplasms**	23	1 (Ref)	
**Adenomas and adenocarcinomas**	572	0.78 (0.39-1.17)	0.158
**Complex epithelial neoplasms**	20	1.48 (0.36-2.60)	0.957
**Complex mixed and stromal neoplasms**	1	NA‡	0.997
**Cystic mucinous and serious neoplasms**	25	0.83 (0.23-1.43)	0.229
**Epithelial neoplasms**	260	0.75 (0.32-1.18)	0.147
**Squamous cell neoplasms**	272	0.75 (0.36-1.14)	0.131
**Year of diagnosis**	1173		
**2010-2013**	832	1 (Ref)	
**2014-2015**	341	348.66 (105.19-592.12)	< 0.001
**Stage_T**	1173		
**T0**	3	1 (Ref)	
**T1**	297	14.45 (0.49-28.40)	0.202
**T2**	252	14.46 (0.49-28.43)	0.205
**T3**	186	15.41 (0.51-30.30)	0.188
**T4**	221	10.54 (0.35-20.72)	0.340
**TX**	214	11.57 (0.35-22.79)	0.327
**Stage_N**	1173		
**N0**	529	1 (Ref)	
**N1**	87	1.68 (0.96-2.40)	0.072
**N2**	349	1.23 (0.83-1.63)	0.373
**N3**	90	1.49 (0.79-2.19)	0.294
**NX**	118	1.07 (0.53-1.60)	0.760
**Surgery status**	1173		
**Not performed**	850	1 (Ref)	
**Performed**	323	1.03 (0.72-1.33)	0.898
**Primary tumor size (0.1 mm)**	1173		
**< 100**	885	1 (Ref)	
**100-199**	22	1.71 (0.50-2.91)	0.682
**≥ 200**	266	1.34 (0.81-1.87)	0.336

* Includes: American Indian/AK native, Asian/ Pacific Islander. † Includes: non-Hispanic white, non-Hispanic black and non-Hispanic Asian and Pacific Islander. ‡ Data could not be calculated. Abbreviations, HR, hazard ratio; Ref, reference; NA, not available; SEER database, surveillance epidemiology and end results database.
